# Redlining, Urban Heat, and Climate Hazards: Impacts on Children’s Cardiometabolic Health

**DOI:** 10.1007/s11524-026-01132-9

**Published:** 2026-08-11

**Authors:** Eun Kyung Lee, Brooks B. Gump, Megan B. Cole, Andrea R. Titus, Darcy A. Freedman, Sadeer Al-Kindi

**Affiliations:** Department of Environmental Studies, College of Environmental Science and Forestry, State University of New York, Syracuse, NY, USA; Department of Public Health, Maxwell School of Citizenship and Public Affairs, Syracuse University, Syracuse, NY, USA; Department of Population Medicine, Harvard Medical School &, Harvard Pilgrim Health Care Institute, Boston, MA, USA; Department of Population Health, New York University Grossman School of Medicine, New York, NY, USA; Mary Ann Swetland Center for Environmental Health & Department of Population and Quantitative Health Sciences, Case Western Reserve University, Cleveland, OH, USA; Houston Methodist DeBakey Heart & Vascular Center, Houston, TX, USA; Weill Cornell Medicine, New York, NY, USA

**Keywords:** Cardiometabolic diseases, Redlining, Climate hazards, Urban heat, Child health

## Abstract

Structural inequities, including historical redlining, have created compounding climate-related vulnerabilities in marginalized communities, with potential implications for pediatric cardiometabolic diseases (CMD). However, the joint effects of redlining, climate risk, and urban heat on pediatric CMD remain poorly understood. Using New York State hospital administrative data (2018–2022), we examined associations between neighborhood redlining, climate risk, urban heat, and CMD-related emergency department (ED) and outpatient visits among children aged < 18 years. Exposures included historical redlining (Home Owners’ Loan Corporation grades C/D vs. A/B), climate risk (high vs. low, based on the Climate Vulnerability Index), and urban heat (high vs. low, based on satellite-derived land surface temperature). Associations were estimated using Poisson regression with interaction terms and subtype-specific analyses. Among 342,220 CMD-related visits in the climate-risk cohort and 9,195 in the urban-heat cohort (mean age, 7.4 years; 49% female), residence in historically redlined neighborhoods was associated with increased rates of CMD-related ED visits (rate ratios [RRs] = 1.15; 95% CI, 1.12–1.18) and outpatient visits (RR = 1.18; 95% CI, 1.17–1.20), with the strongest associations observed for comorbidity, hypertension, and type 1 diabetes (T1D). High-climate risk was associated with modestly lower overall rates of CMD-related ED visits (RR = 0.95; 95% CI, 0.94–0.96) and outpatient visits (RR = 0.96; 95% CI, 0.96–0.97), although elevated risks remained evident for selected CMD subtypes (e.g., hypertension, obesity, T1D). In the urban-heat cohort, most associations were not statistically significant (RRs = 1.00–1.03), except for outpatient visits for T1D, where higher urban heat exposure was associated with increased risk (RR = 1.28; 95% CI, 1.04–1.57). Co-exposure to historical redlining and high-climate-risk was associated with a modest increase in CMD-related ED visits (RR = 1.04; 95% CI, 1.01–1.07), whereas no significant associations were observed for co-exposure to redlining and urban heat (RR = 1.08; 95% CI, 0.78–1.49). Associations for outpatient visits were attenuated in both analyses. These findings suggest that redlining may be a stronger determinant of pediatric CMD healthcare utilization than climate-related hazards alone, highlighting the enduring health consequences of structural inequities and underscoring the need for targeted, climate-adaptive interventions, especially in historically redlined communities.

## Introduction

The prevalence of pediatric cardiometabolic diseases (CMDs), including obesity, diabetes, and cardiovascular conditions, has risen substantially in the United State (U.S.) [1–3]. Over the past three decades, childhood obesity rates have doubled, while adolescent rates have tripled. Currently, approximately 19.7% of U.S. children aged 2–19 are classified as obese, with rates projected to continue increasing [4, 5]. Pediatric diabetes and cardiovascular conditions have likewise increased over time [6]. Alongside well-established neighborhood vulnerabilities associated with CMD risk, such as limited access to green space, healthy foods, and healthcare, emerging evidence suggests that climate-related exposures and neighborhood environments may further exacerbate pediatric cardiometabolic risk [6, 7].

Natural disasters, including extreme temperatures, flooding, hurricanes, and wildfires, are increasing in both frequency and severity [8]. These climate events have been linked to elevated CMD risk, with adverse effects that may persist for months or even years after a disaster. Several mechanisms may underlie this relationship, including direct exposures such as extreme heat (≥ 95 °F) and climate-driven air pollution (e.g., wildfire smoke), as well as indirect pathways, including infrastructure damage, disrupted access to healthcare, particularly for chronic conditions such as hypertension, and psychological stress, which can elevate cortisol levels and blood pressure [9–11].

Historically disadvantaged neighborhoods, especially those shaped by disinvestment and discriminatory housing policies such as redlining, also bear a disproportionate burden of weather-related disasters due to inadequate infrastructure and limited adaptive capacity [12–14]. Although redlining, a racially discriminatory lending practice prevalent in the 1930s–1940s, was formally outlawed in the 1960 s, its legacy persists in the built environment and continues to contribute to adverse health outcomes, including CMDs [13, 15–17]. Recent research continues to illuminate that formerly redlined areas experience greater exposure to climate-related hazards, including elevated temperatures, increased flood risk, and more frequent power outages, exacerbating health inequities [18–20]. These overlapping exposures highlight the importance of evaluating both redlining and climate-related risks together, as the health impacts of, and responses to climate hazards may differ according to structural differences in neighborhood resources and resilience [21–23].

Children represent a particularly vulnerable segment of the population to climatic, environmental, and social conditions, owing to their developing physiology, immature thermoregulatory capacity, and higher surface area-to-mass ratios, which together heighten their susceptibility to heat- and other climate-related physiological stress [24]. Early onset of clinical risk factors, such as diabetes and hypertension, substantially increases the risk of adult cardiovascular disease, the leading cause of death in the U.S., underscoring the importance of early intervention during this developmental stage. The present study examines the independent and joint effects of climate risks, urban heat, and residence in historically redlined neighborhoods on pediatric CMD-related healthcare utilization in New York State (NYS) from 2018 to 2022.

## Methods

### Study Population and Outcomes

**Fig. 1:**
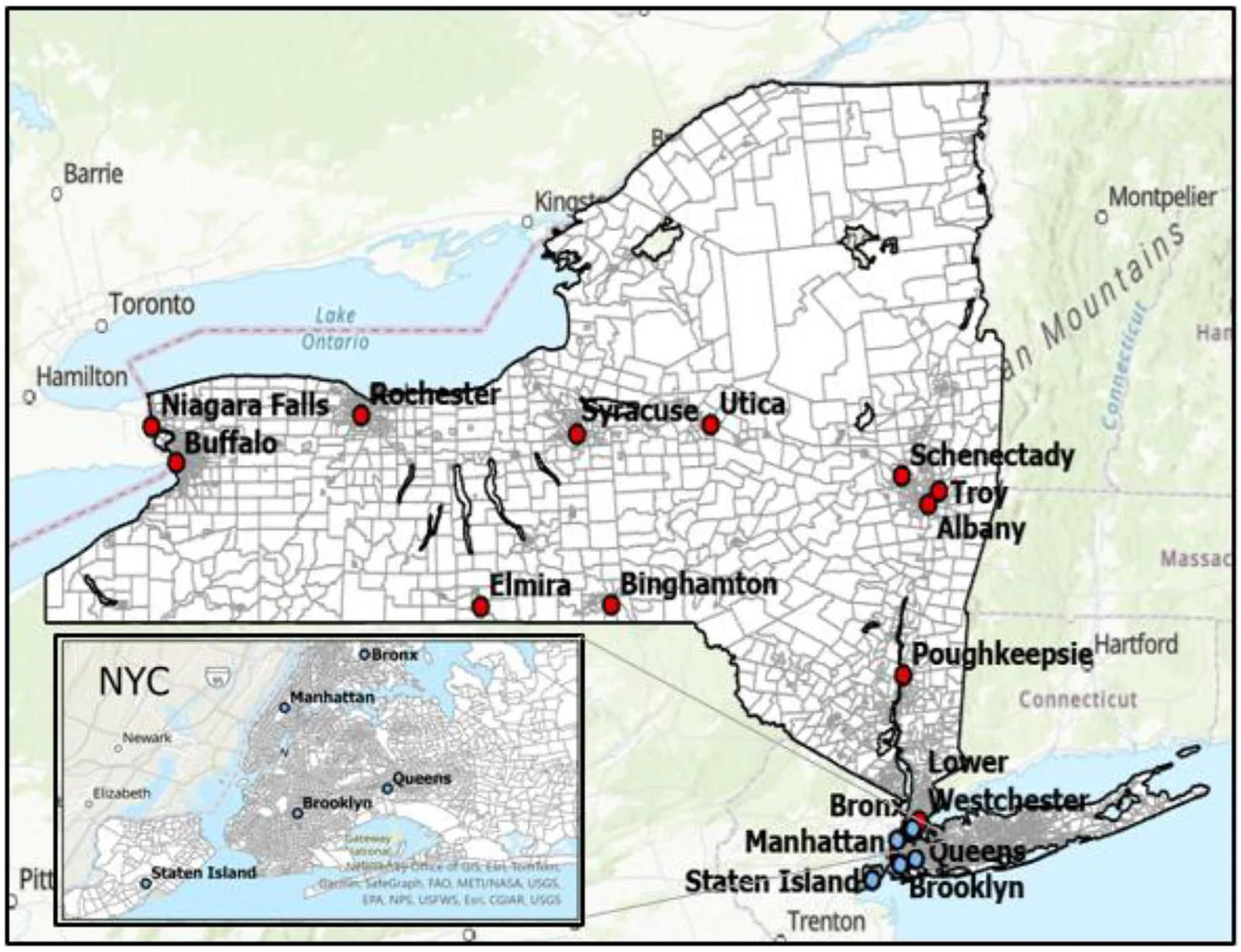
Study regions (13 cities) across New York State. The five boroughs of New York City (Bronx, Brooklyn, Manhattan, Queens, Staten Island), shown as separate regions (blue dots) here, were considered a single city for the analyses

This study included all CMD-related emergency department (ED, including urgent care) and outpatient visits among children aged 0–17 residing in areas formerly graded by the Home Owners’ Loan Corporation (HOLC) across 13 NYS cities from 2018 to 2022. For analysis, the five boroughs of New York City were treated as a single city (Fig. 1). CMDs were categorized into seven subtypes and examined accordingly, including primary diagnoses of comorbidity (e.g., two or more CMDs), hypertension, hyperlipidemia, overweight/obesity, type 1 and type 2 diabetes (T1D, T2D), and cardiovascular diseases (acute myocardial infarction, atrial fibrillation, congestive heart failure, stroke/transient ischemic attack, and ischemic heart disease), as identified using ICD-10 codes. Visits were aggregated annually and monthly at the census-tract level for each HOLC-graded city, and the total number of children in each census tract was used as the denominator [25].

Data were obtained from the New York State Department of Health’s Statewide Planning and Research Cooperative System (SPARCS), which captures approximately 95% of hospital admission and discharges in the state [26]. SPARCS provides individual-level sociodemographic data (age, sex, race/ethnicity), insurance type, residential address, and visit-level clinical information. Patients with unknown race/ethnicity were excluded. Residential addresses were geocoded and aggregated to the census tract level using ArcGIS Pro. The study was deemed exempt by the Syracuse University Institutional Review Board, with a waiver of informed consent, as the data were de-identified and derived from an existing administrative database.

### Exposure Assessment

Among children with at least one CMD-related visit to a NYS hospital between 2018 and 2022, we first compared those residing in historically redlined versus non-redlined areas. In the 1930 s and 1940 s, the Home Owners’ Loan Corporation (HOLC), working within broader public–private systems of mortgage finance and real estate appraisal, produced residential security maps for more than 239 U.S. cities. These maps graded neighborhoods according to perceived lending risk based on housing conditions, neighborhood amenities, and, critically, racial and ethnic composition [27–29]. Neighborhoods with larger Black, immigrant, and low-income populations were disproportionately classified as “hazardous” (redlined), reinforcing racialized disinvestment and residential segregation. Although HOLC maps did not independently create discriminatory housing practices, they reflected and institutionalized existing systems of racialized housing exclusion and unequal capital investment.

HOLC grades, now commonly used as a spatial proxy for historical redlining because of their close spatial alignment with formerly redlined neighborhoods and their digitized availability, were obtained from the University of Richmond’s Mapping Inequality database [30]. Consistent with Swope et al. [29], we emphasize that HOLC grades are not synonymous with redlining itself; rather, they represent one manifestation of structural racism and capture broader patterns of institutionalized disinvestment and discriminatory urban development. Additionally, Faber’s comparative analysis of cities that underwent HOLC appraisal and those that did not demonstrated that HOLC appraisal was associated with elevated levels of residential segregation that have persisted to the present day, thereby elucidating a plausible pathway through which historical redlining may continue to shape contemporary outcomes [31]. Accordingly, neighborhoods were classified as A (green, “best”), B (blue, “still desirable”), C (yellow, “definitely declining”), or D (red, “hazardous”) based on the aforementioned metrics.

HOLC maps were spatially joined to the most recently available U.S. Census TIGER/Line tracts using ArcGIS Pro and tracts spanning multiple grades were assigned the predominant grade (≥ 50% of the land area) following the method of Krieger et al. [32]. C- and D-graded tracts were categorized as “redlined,” and A- and B-graded tracts as “non-redlined” to ensure sufficient sample size and enhance interpretability.

Given the increasing frequency of climate-related disasters, our secondary exposure focused on climate risk using two indicators (eFigure 1). First, we used the climate change risk domain of the Climate Vulnerability Index (CVI), developed by the U.S. Environmental Defense Fund and Texas A&M University [33]. This domain incorporates health impacts (e.g., disaster- or temperature-related deaths), social and economic costs (e.g., disaster-related financial stress), and extreme weather events (e.g., extreme heat, flooding, storms, droughts, wildfires), with scores ranging from 0 (low risk) to 1 (high risk). To ensure an adequate sample size and enhance interpretability, census tracts were classified as “high” or “low” climate risk based on city- and HOLC-grade-specific median CVI values.

Second, we assessed urban heat, the leading cause of weather-related morbidity and mortality [8]. Urban heat was analyzed separately from the CVI to capture temporally specific exposure, avoid double-counting the extreme heat component, and better evaluate neighborhood-level effects on CMDs. Mean land surface temperatures (LSTs), based on Landsat satellite data (30 × 30 m resolution), were calculated using Zonal Statistics in ArcGIS Pro, and tracts were classified as “high” or “low” urban heat exposure based on city-, HOLC-grade-, and month-specific median LST values. Subsequently, CMD-related visits were linked annually to census tract-level CVI and, during the summer months of each year (June–August, 2018–2022), to census tract-level LST [34].

### Other Covariates

Covariates included child-level age, race/ethnicity, and sex from the SPARCS database [26]. To account for neighborhood context, we adjusted for four domains: (a) socioeconomic factors—poverty rate, vehicle access, population density, hospital proximity, and local homicide and gun violence rates [33]; (b) food environment—food insecurity prevalence and healthy food access [35, 36]; (c) environmental pollution—proximity to high-traffic roads (vehicles per meter within 500 m), industrial and treatment/storage/disposal facilities (TSDFs), and diesel particulate matter (PM_2.5_, μg/m^3^), all linked to cardiovascular risk [37]; and (d) built environment—walkability, cooling center availability, and green space coverage, measured by the Normalized Difference Vegetation Index (NDVI; range − 1 to + 1, with values near − 1 indicating non-vegetated areas [34], given its relevance to physical activity and urban heat mitigation [38–40].

### Statistical Analysis

The unit of analysis was the annual and monthly number of ED and outpatient visits for pediatric CMD patients per census tract. Poisson regression models were conducted to estimate rate ratios (RRs) and 95% confidence intervals (CIs) for associations between CMD-related healthcare utilization and climate-related exposures (climate risk, urban heat) in the context of redlining, adjusting for relevant covariates and incorporating an offset for child census population within each tract.

To assess independent effects, we compared CMD visit rates among children living in redlined versus non-redlined neighborhoods, as well as those experiencing high versus low-climate-risk or high versus low-urban-heat exposure, in unadjusted models (model 1), models adjusted for the child’s age, sex, and race/ethnicity (model 2), and models additionally adjusted for covariates (model 3) [41–45]. Joint effects were evaluated using interaction terms (e.g., redlining × high-climate-risk, redlining × high-urban-heat), using non-redlined and low-risk areas as reference categories, where appropriate. All models were evaluated for both overall CMDs and specific CMD subtypes, as follows:

$$\text{log}\;(\text{E}(\text{CMD}\;\text{cases}|\text{X}))=\beta_{0}\;{\text{redlining}(\text{exposure}}_{1})+\beta_{1}\;{\text{high}\;\text{climate}\;\text{risk}\;\text{or}\;\text{urban}\;\text{heat}\;(\text{exposure}}_{2})+\beta_{2}\;\text{redlining}*\text{high}\;\text{climate}\;\text{risk}\;\text{or}\;\text{urban}\;\text{heat}+\beta_{3}\;\text{sex}+\beta_{4}\;\text{age}+\beta_{5}\;\text{race}/\text{ethnicity}+\text{offset}\;(\text{log}(\text{population}))+\beta_{\text{n}}\;\text{covariates}$$

As sensitivity analyses, we assessed differences across time periods (pre-pandemic, 2018–2019 vs. pandemic, 2020–2022), to account for shifts in healthcare access, indoor exposures, stress, physical activity, and climate-related impacts before and during COVID-19 pandemic. All *p*-values were two-sided, with statistical significance set at *α* < 0.05. Analyses were conducted using SAS version 9.4.

## Results

### Study Population

The final analytic sample included 342,220 pediatric CMD-related healthcare visits from 2018 to 2022, comprising 41,447 (12.1%) ED visits and 307,450 (87.9%) outpatient visits. Of these, 258,220 visits (83.3%) occurred in historically redlined areas and 57,000 (16.7%) in non-redlined areas. Overall, 40% of healthcare visits were from Hispanic children, 18% from non-Hispanic Black, 9% from non-Hispanic White, 7% from children of other racial groups, and 5% from non-Hispanic Asian children (Table 1). Approximately 40% were under age 5, 20% were aged 5–9, 22.4% aged 10–14, and 17.1% aged 15–17. The sex distribution was nearly equal (51% male, 49% female). Most of these visits were by children with private insurance (58.4%), followed by Medicaid/Child Health Plus (CHP) (27.5%). Obesity was the most common CMD subtype (72.5%), followed by type 1 diabetes (T1D) (8.0%) and hypertension (6.3%). A subsample of 9,165 CMD-related visits occurring between June and August was analyzed to assess associations with urban heat (eTable 1).

### Climate Risk Scores and Urban Heat Exposure by Redlining Status

**Fig. 2:**
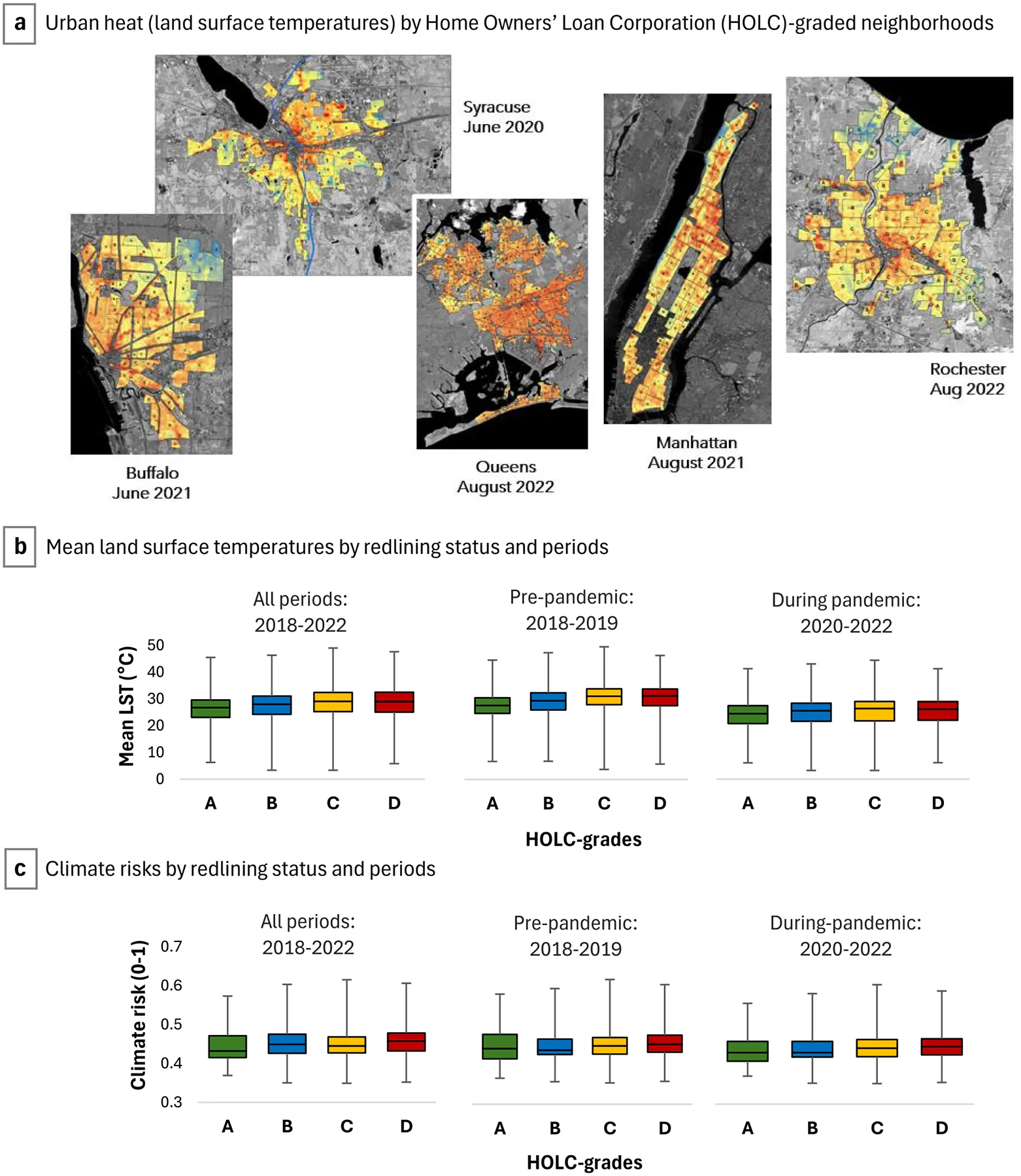
Spatio-temporal patterns of urban heat and climate risks by redlining status across New York State. Selected maps illustrating urban heat or mean land surface temperatures (LST) by Home Owner’s Loan Corporation-graded areas across New York State (**a**); Boxplots showing urban heat and climate risk measures across time periods and by redlining status (**b**, **c**). *Note:* In the maps, red represents higher LSTs, and blue represents lower LSTs. The error bars in the boxplots, indicate the minimum and maximum values, while the lines in the box plots represent the 25th, median, and 75th percentile values

Figure 2 displays selected maps and temporal trends in climate risk scores and urban heat. Across the study period (2018–2022), climate risk scores remained relatively stable over time, with redlined areas consistently exhibiting modestly higher scores than non-redlined areas. For urban heat measures, redlined areas (C- and D-graded areas) recorded higher land surface temperatures (LSTs), with average values ranging from 23.5 to 31.2 °C. In contrast, non-redlined areas exhibited lower average LSTs, ranging from 22.2 to 29.5 °C, approximately 1.3 to 1.7 °C lower than those in redlined areas. Mean LSTs declined during the pandemic years (2020–2022), averaging 26.3 °C, compared with 29.6 °C during the pre-pandemic period (2018–2019).

### Redlining and CMD Healthcare Visits

**Fig. 3:**
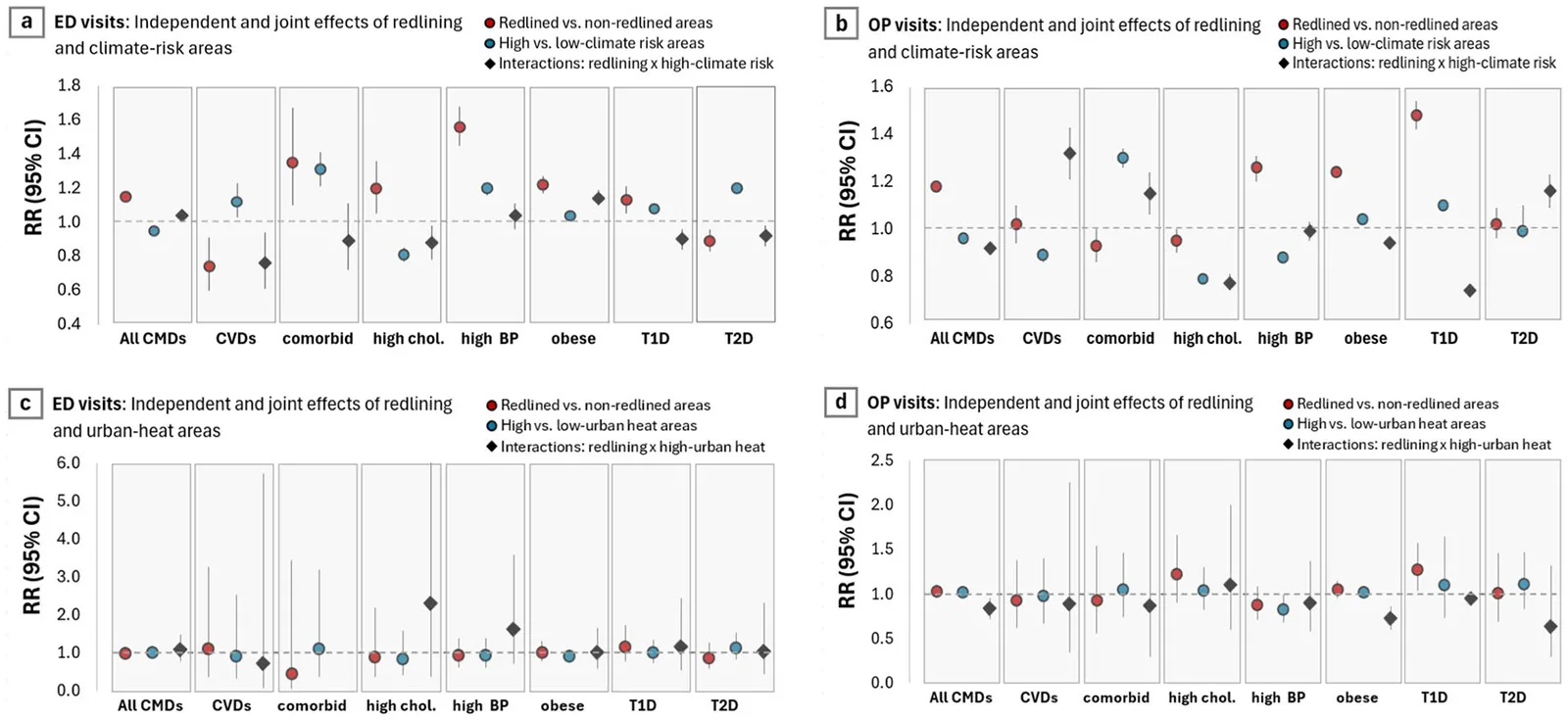
Residential exposure effects of redlining and climate-related hazards on pediatric cardiometabolic healthcare visits in New York State, 2018–2022. ED and outpatient visits related to the independent and joint effects of residence in redlined and high-climate risk areas (**a**, **b**); and ED and outpatient visits related to the independent and joint effects of residence in redlined and high-urban-heat areas (**c**, **d**). *Abbreviations:* ED, emergency department visits; OP, outpatient visits; RR, rate ratios; CI, confidence intervals; CMD, cardiometabolic disorders; CVD, cardiovascular disease; chol., cholesterol; BP, blood pressure; T1D and T2D, type 1 and 2 diabetes. *Note:* All results were based on fully adjusted models

Poisson regression analyses using independent-effects models indicated that, among 342,220 CMD-related healthcare visits evaluated for climate-related vulnerabilities, residence in historically redlined neighborhoods was associated with a 15% higher risk of CMD-related ED visits (95% CI, 1.12–1.18) and a 18% higher risk of CMD-related outpatient visits (95% CI, 1.17–1.20) compared with peers in non-redlined areas (Fig. 3a,). While overall CMD-related healthcare utilization was consistently higher among children in historically redlined neighborhoods, subtype-specific analyses revealed important heterogeneity in both the magnitude and statistical significance of these associations. For ED visits, children residing in redlined neighborhoods had 35% higher comorbidity-related visits (95% CI, 1.10–1.67), 20% higher high cholesterol-related visits (95% CI, 1.05–1.36), 56% higher hypertension-related visits (95% CI, 1.45–1.68), 22% higher obesity-related visits (95% CI, 1.17–1.27), and 13% higher type 1 diabetes (T1D)-related visits (95% CI, 1.05–1.21) compared to those in non-redlined areas (Fig. 3a). For outpatient visits, we observed 26% higher hypertension-related visits (95% CI, 1.20–1.31), 24% higher obesity-related visits (95% CI, 1.23–1.26), and 48% higher T1D-related visits (95% CI, 1.42–1.54). Cardiovascular disease (CVD) and type 2 diabetes (T2D) showed 2% higher associations, but did not reach statistical significance (Fig. 3b).

Stratified analyses by pandemic periods further showed that higher CMD-related ED visit rates among children in redlined neighborhoods, compared with those in non-redlined areas, remained elevated both before and during the pandemic (RRs = 1.13–1.15). Across CMD subtypes, the magnitude of associations was generally constant or higher during the pandemic, with the most notable increase observed for comorbidity-related ED visits, which rose from 17% higher before the pandemic (95% CI, 0.94–1.46) to 405% higher during the pandemic (95% CI, 2.28–11.2). In contrast, for outpatient visits, the magnitude of associations decreased during the pandemic, potentially indicating lower CMD-related healthcare utilization (eTable 2).

Among 9165 CMD-related healthcare visits evaluated for urban heat exposure during summer, CMD-related ED visits did not differ between children in redlined and non-redlined neighborhoods (RR = 1.00; 95% CI, 0.85–1.18), though a modest, non-significant 3% increase was observed in outpatient visits (95% CI, 0.96–1.10) (Fig. 3c). Subtype analyses showed that children in high-heat neighborhoods had a 28% higher risk of T1D-related ED visits (95% CI, 1.04–1.57) compared to those in cooler areas. Stratification by pandemic periods revealed elevated, but non-significant, risks for several CMD-related ED and outpatient visits before and during the pandemic (eTable 3).

### Climate Risks and CMD Healthcare Visits

Analyses by climate-risk designation indicated that residence in high-risk neighborhoods—defined as areas prone to flooding, hurricanes, wildfires, or other extreme weather events—was not associated with increased CMD-related ED (RR = 0.95; 95% CI, 0.94–0.96) or outpatient visits (RR = 0.96; 95% CI, 0.96–0.97) (Fig. 3a,). However, subtype-stratified analyses showed elevated risks across six CMD subtypes, including 12% higher CVD-related ED visits (95% CI, 1.03–1.23), 31% higher comorbidity-related ED visits (95% CI, 1.21–1.41), 20% higher hypertension-related ED visits (95% CI, 1.16–1.23), 4% higher obesity-related ED visits (95% CI, 1.02–1.06), 8% higher T1D-related ED visits (95% CI, 1.08–1.09), and 20% higher T2D-related ED visits (95% CI, 1.17–1.23). For outpatient visits, three subtypes were associated with increased risk: 30% higher comorbidity-related visits (95% CI, 1.26–1.34), 4% higher obesity-related visits (95% CI, 1.03–1.04), and 10% higher T1D-related visits (95% CI, 1.08–1.12). Stratification by COVID-19 period revealed that, residents in high-climate risk areas generally had lower overall ED and outpatient utilization for all CMDs both before and during the pandemic (RRs = 0.94–0.97) (eTable 2). However, important differences emerged by CMD subtypes. For ED visits, high-risk areas were associated with higher utilization for CVDs, comorbidity, hypertension, obesity, and T1D before the pandemic, and these associations largely persisted during the pandemic, although the magnitude of associated attenuated for most CMD subtypes. Comorbidity showed the strongest increase during the pandemic (RR = 1.63; 95% CI, 1.16–2.30). T1D-related ED visits also remained elevated, while T2D shifted from lower utilization pre-pandemic to no significant difference during the pandemic.

For outpatient visits, high-risk areas were associated with lower utilization for CVDs, hyperlipidemia, hypertension, and T2D in both periods. In contrast, comorbidity and obesity were consistently associated with higher outpatient utilization. Notably, T1D outpatient visits changed from lower utilization pre-pandemic to substantially higher utilization during the pandemic (RR = 1.20; 95% CI, 1.17–1.22). Overall, the pandemic appeared to modify healthcare utilization patterns in high-climate risk areas, particularly for comorbidity, T1D, and T2D.

### Urban Heat Exposure and CMD Healthcare Visits

Urban heat exposure analyses demonstrated no significant associations with overall CMD-related ED visits (RR = 1.00; 95% CI, 0.87–1.13) or outpatient visits (RR = 1.02; 95% CI, 0.97–1.08). Subtype-stratified analyses revealed a 24% increase in T1D-related outpatient visits among children residing in high-urban-heat areas (95% CI, 1.06–1.46), whereas other CMD subtypes showed reduced or non-significant associations (Fig. 3). Pandemic-period stratification indicated that, unlike climate-risk areas, urban heat exposure effects were more pronounced during the pandemic: overall outpatient visits increased by 6% (95% CI, 1.00–1.13), and diabetes-related outpatient visits increased by 31% (95% CI, 1.12–1.53), suggesting persistent influence of urban heat on CMD-related healthcare utilization despite broader healthcare disruptions (eTable 3).

### Joint Effects of Redlining, Climate Risk, and Urban Heat Exposure and CMD Healthcare Visits

Interactive analyses of redlining and climate-related risks indicated that the associations for both ED and outpatient visits were either modestly higher (ED, RR = 1.04; 95% CI, 1.01–1.07) or attenuated (outpatient, RR = 0.92; 95% CI, 0.91–0.93). By CMD subtype, only obesity-related ED visits (RR = 1.14, 95% CI, 1.10–1.19) were associated with increased risk from joint exposure to redlining and high climate risks. Among CMD subtypes, CVD-related (RR = 1.32; 95% CI, 1.21–1.43), comorbidity-related (RR = 1.15; 95% CI, 1.06–1.24), and T2D-related outpatient visits (RR = 1.16; 95% CI, 1.09–1.23) were associated with increased risks (Fig. 3). When stratified by pandemic period, hypertension-related ED visits were associated with a 20% increased risk before the pandemic (RR = 1.20; 95% CI, 1.07–1.35), but not during the pandemic (RR = 0.97; 95% CI, 0.89–1.07). By contrast, associations for other CMD subtypes, including CVD, obesity, and T2D, persisted both before and during the pandemic, although only CVD-related outpatient visits during the pandemic remained statistically significant (RR = 1.26; 95% CI, 1.22–1.51) (eTable 4).

Examination of redlining and urban heat interactions suggested elevated associations for several CMD subtypes (e.g., high cholesterol, hypertension, T1D, and T2D); however, these estimates did not reach statistical significance, showing a similar pattern before and during the pandemic.

## Discussion

In this large, population-based study spanning New York State from 2018 to 2022, we examined 342,220 pediatric CMD-related visits in the climate risk cohort and 9195 visits in the urban heat cohort to evaluate the independent and joint effects of residence in historically redlined, high-climate-risk, and urban-heat-exposed areas on the burden of CMD among children. Overall, we observed the strongest and most consistent increases in healthcare utilization among children residing in historically redlined neighborhoods compared with non-redlined areas, with more modest associations observed for high-climate-risk exposure and limited evidence for urban heat exposure. We also observed substantial heterogeneity across CMD subtypes, which may inform more targeted, condition-specific clinical and public health interventions. To our knowledge, this study represents the first statewide analysis leveraging patient-level healthcare utilization data in a pediatric population, a particularly vulnerable group, to elucidate the combined impacts of neighborhood redlining, climate-related hazards, and urban heat on cardiometabolic health.

As hypothesized, children residing in historically redlined neighborhoods had higher rates of CMD-related ED and outpatient visits compared to those in non-redlined areas. When disaggregated by CMD subtype, redlined residence was associated with an increased risk of comorbidity-, cholesterol-, hypertension-, obesity-, and T1D-related visits. This suggests that redlining may influence specific CMD pathways, possibly through limited access to healthy food or green space [17, 46, 47]. These subtype-specific associations were strongest prior to the COVID-19 pandemic, except for certain CMD subtypes, including comorbidity-related ED visits, suggesting that the pandemic may have disrupted care-seeking behaviors and patterns of healthcare utilization.

Regarding climate risk, high-risk areas was not consistently associated with overall CMD outcomes, despite prior evidence linking extreme weather events to adverse health outcomes [48]. However, stratified analyses revealed higher rates of ED visits for several CMD subtypes, including CVD-, comorbidity-, hypertension-, obesity-, T1D-, and T2D-related conditions, as well as increased outpatient visits for comorbidity-, obesity-, and T1D-related conditions. Notably, comorbidity-related ED visits and T1D-related outpatient visits were more pronounced during the pandemic than in the pre-pandemic period. These findings suggest that the health impacts of climate-related stressors may vary across CMD subtypes and be modified by broader contextual factors, such as the COVID-19 pandemic. The concurrent burden of climate-related hazards and pandemic-related disruptions may have created compounded challenges for disease management and healthcare access, particularly following extreme weather events [49]. At the same time, shifts in healthcare-seeking behavior during the pandemic, including greater reliance on telehealth and reductions in in-person care utilization [50], may have influenced observed patterns of healthcare use. Collectively, these findings underscore the need for disaster preparedness and healthcare system resilience to ensure continuity of care for children with chronic conditions, particularly those with hypertension and multiple comorbidities, during overlapping public health and climate-related emergencies, while recognizing that healthcare utilization may not fully reflect the underlying burden of CMDs in structurally disadvantaged populations due to barriers to healthcare access.

Similarly, while overall CMD-related visits were not significantly associated with urban heat exposure, we found a strong and specific link with T1D. Children residing in high-urban-heat neighborhoods experienced a 24% increase in T1D-related outpatient visits, an effect absent before the pandemic but intensified during the pandemic. Unlike the impacts of cumulative climate risks, this finding supports emerging evidence that heat exposure may uniquely affect diabetes by impairing glycemic control and increasing insulin demand, particularly among children confined to high-heat environments due to both the pandemic and the characteristics of their neighborhoods. Such conditions can dysregulate blood glucose levels through insulin degradation, compounded by pandemic-related stressors, underscoring the need for targeted heat-mitigation strategies for the specific segment of children disproportionately affected [51].

Joint exposure analyses of redlining and climate-related risks showed largely modest or attenuated associations with CMD-related healthcare utilization overall. However, subtype-specific analyses revealed increased risks of obesity-related ED visits and CVD-, comorbidity-, and T2D-related outpatient visits among children residing in redlined neighborhoods with high climate risk, suggesting that structural disadvantage and climate-related stressors may jointly exacerbate specific CMD outcomes [52]. Potential mechanisms include direct exposure to climate hazards, stress-related physiologic responses, prenatal environmental exposures, and disruptions in healthcare access and health-seeking behaviors [53]. Stratified analyses further indicated temporal heterogeneity, with elevated hypertension-related ED visits observed prior to the pandemic, whereas CVD- and obesity-related risks were more pronounced during the pandemic period.

Taken together, our findings suggest that co-exposure to redlining and elevated climate risk may be associated with increased CMD-related healthcare utilization. However, these associations varied substantially across CMD subtypes, underscoring the importance of subtype-specific analyses and the careful interpretation of aggregate estimates. Overall estimates may mask important vulnerabilities that could inform more targeted clinical and public health interventions. For instance, elevated rates of CVD-related ED visits in redlined areas, together with the observed effects of co-exposure to climate hazards, may reflect the compounded influence of structural inequities, including neighborhood disinvestment and the lack of health-promoting community resources. These disadvantages may accumulate across the life course and extend to the prenatal period, as maternal exposure to climate-related hazards has been linked to adverse cardiovascular outcomes in offspring [53]. Additionally, the more nuanced associations observed for climate and heat exposures highlight the need for targeted public health interventions addressing specific CMD subtypes in pediatric populations, while also accounting for disaster-related contextual factors, such as the COVID-19 pandemic and increased indoor confinement, which may influence cardiometabolic risk. Further analyses comparing cities within NYS, including rural vs. urban areas, along with more extensive analyses incorporating daily temperature exposures and racial and ethnic disparities, are warranted to validate these findings given the substantial variability in demographic and environmental characteristics [54].

This study has some limitations to consider when interpreting its findings. First, the dataset primarily consisted of hospital visits, likely capturing only the most severe cases or individuals who regularly use healthcare services, billed cases, or only those who have access to care. Nevertheless, these data provide valuable insight into real-world patterns of CMD-related healthcare use during a period of heightened vulnerability among children. Second, LST and climate risk metrics were used as area-level proxies. The CVI, based on data from a single year, may not adequately capture individual-level or temporally specific exposures. Future research should incorporate longer time spans and more granular, disaster-specific data to validate these findings. Third, healthcare utilization patterns were significantly affected by the COVID-19 pandemic, potentially confounding the observed associations between environmental exposures and CMD outcomes—a key reason for stratifying analyses by time periods (e.g., pre- versus during COVID-19). These insights should be carefully considered when interpreting the findings. Moreover, the limited sample sizes, particularly in analyses of CMD interactions and subtypes, may have increased the risk of overfitting and reduced the statistical power, representing an additional limitation of the study. Future studies could address this limitation by expanding the dataset to include additional years or months. Finally, the potential for residual confounding from unmeasured social or environmental factors, including access to healthcare and care-seeking behavior, both of which may be shaped by factors including health insurance coverage, transportation availability, and caregivers’ work-related scheduling constraints, should be taken into account when interpreting the findings of this study, as these factors may influence utilization-based outcomes.

## Conclusion

In this multi-year, co-exposure study of pediatric cardiometabolic health in New York State, we observed higher CMD-related healthcare utilization in redlined compared with non-redlined areas, with modest increases in neighborhoods characterized by high climate risk and urban heat, and additional elevations under co-exposure with redlining. Disease-specific analyses revealed pronounced vulnerabilities, particularly for CVDs, cholesterol, obesity, hypertension, diabetes, and pandemic-related disruptions. These findings underscore the urgent need for targeted and timely public health strategies and preparedness efforts, particularly for specific CMD subtypes, while also addressing historical and contemporary environmental injustices that contribute to structurally embedded vulnerability.

## Supplementary Material

Supplementary file

**Supplementary Information** The online version contains supplementary material available at https://doi.org/10.1007/s11524-026-01132-9.

## Figures and Tables

**Table 1:** Pediatric healthcare visit and census tract characteristics by redlining status in New York State, 2018–2022

Characteristics	Residential redlining status, No. (%)		
	Total (A-D grades)^a^	Non-redlined areas (A/B grades)^a^	Redlined areas (C/D grades)^a^
	(*n* = 342,220; 100%)	(*n* = 57,000; 16.7%)	(*n* = 285,220; 83.3%)
Individual-level sociodemographic characteristics			
Emergency department visits	41,447 (12.1)	6984 (16.9)	34,463 (83.1)
Outpatient services	300,773 (87.9)	50,016 (16.6)	250,757 (83.4)
Disease type			
Cardiovascular diseases^b^	6696 (2.0)	1279 (2.2)	5417 (1.9)
Comorbidity	7166 (2.1)	1211 (2.1)	5955 (2.1)
Hyperlipidemia or high cholesterol	14,928 (4.4)	2743 (4.8)	12,185 (4.3)
Hypertension	21,568 (6.3)	4332 (7.6)	17,236 (6.0)
Obesity	248,217 (72.5)	39,728 (69.7)	208,489 (73.1)
Type 1 diabetes (T1D)	27,474 (8.0)	5062 (8.9)	22,412 (7.9)
Type 2 diabetes (T2D)	16,171 (4.7)	2645 (4.6)	13,526 (4.7)
Race and ethnicity			
Black, non-Hispanic	65,426 (17.9)	10,231 (17.9)	55,195 (19.4)
Hispanic, any race	140,801 (40.1)	22,750 (40.1)	117,218 (41.3)
White, non-Hispanic	20,096 (9.9)	5621 (9.9)	14,475 (5.1)
Asian, non-Hispanic	15,179 (4.9)	2778 (4.9)	12,401 (4.3)
Other race, non-Hispanic^c^	26,936 (7.0)	4006 (7.0)	22,930 (8.0)
Unknown^d^	73,782 (20.1)	11,480 (20.1)	62,302 (21.8)
Age, mean (SD)	7.4 (5.9)	7.4 (5.9)	7.4 (5.9)
Age group, y			
Less than 5	137,359 (40.1)	22,919 (40.2)	114,440 (40.1)
5 to 9	69,647 (20.4)	11,640 (20.4)	58,007 (20.3)
10 to 14	76,621 (22.4)	12,675 (22.2)	63,946 (22.4)
15 to 17	58,593 (17.1)	9766 (17.1)	48,827 (17.1)
**Sex**			
Female	166,204 (48.6)	27,722 (48.6)	138,482 (48.6)
Male	175,968 (51.4)	29,271 (51.4)	146,697 (51.4)
Insurance coverage type			
Private	151,875 (58.4)	25,114 (57.8)	126,761 (58.5)
Medicaid/CHP	71,679 (27.5)	10,325 (23.8)	61,354 (28.3)
Self-pay	9040 (3.5)	1703 (3.9)	7337 (3.4)
Other/unknown	27,660 (10.6)	6296 (14.5)	21,364 (9.9)
Census tract-level characteristics^e^			
Census tracts	2737 (100)	624 (22.8)	2113 (77.2)
Cooling centers	817 (100)	130 (15.9)	687 (84.1)
Below poverty, % mean (SD)	67.9 (28.0)	67.3 (25.7)	75.5 (24.0)
Climate risk score, median (IQR)	0.45 (0.43, 0.48)	0.51 (0.47, 0.54)	0.54 (0.49, 0.57)
Diesel PM_2.5_ (μg/m^3^), median (IQR)	1.70 (0.95, 2.11)	1.73 (1.51, 2.18)	1.87 (1.51, 2.21)
Food insecurity, mean (SD)	13.1 (3.75)	13.8 (3.2)	14.0 (3.4)
Green space (NDVI), median (IQR)^f^	0.22 (0.18, 0.30)	0.21 (0.16, 0.27)	0.22 (0.17, 0.27)
HHs with no vehicle, % mean (SD)	46.1 (28.2)	52.8 (24.2)	56.1 (22.6)
Homicide rate, mean (SD)	4.3 (1.8)	4.6 (1.7)	4.5 (1.7)
Proximity to hazardous sites, mean (SD)^g^	59.8 (58.7)	59.3 (45.5)	74.4 (57.8)
Proximity to traffic roads, mean (SD)^h^	2395.0 (3 235.0)	2341.7 (2338.2)	2821.3 (3543.2)
Proximity to hospital, mean (SD)	2.7 (2.7)	2.1 (1.6)	2.1 (1.5)
Urban heat, LST (°C), median (IQR)	28.5 (24.6, 31.7)	27.4 (23.7, 30.8)	29.1 (25.9, 32.3)
Walkability index, mean (SD)^i^	25.3 (13.1)	83.3 (19.8)	85.3 (16.6)

*CMD* cardiometabolic diseases, *HOLC* Home Owners’ Loan Corporation, *CHP* Children’s Health Plus, *LST* land surface temperature, *PM* particulate matter, *NDVI* Normalized Difference Vegetative Index, *HH* household

a HOLC grades represent historical neighborhood classifications used in residential security maps: grade A (green) denotes “best” areas, grade B (blue) “still desirable,” grade C (yellow) “declining,” and grade D (red) “hazardous” areas

b Cardiovascular diseases included acute myocardial infarction, atrial fibrillation, congestive heart failure, stroke or transient ischemic attack, ischemic heart disease

c Other race included American Indian or Alaska Native, Native Hawaiian or other Pacific Islander, or other race

d Unknown included children who self- or guardian reported as unknown or those recorded but reported no ethnicity

e Data sources included the American Community Survey (ACS) and the U.S. Environmental Protection Agency (EPA), obtained through the EJScreen tool

f NDVI representing green space, range from − 1 to + 1, close to + 1 indicating higher level of green space and values closer to − 1 indicating non-vegetative areas

g Hazardous waste proximity was based on count of hazardous waste management facilities within 5 km (or nearest one beyond 5 km), each divided by distance in km (sites/km)

h Traffic proximity and volume was based on count of vehicles (annual average daily traffic) at major roads within 500 m, divided by distance in meters (vehicles/m)

i The Walkability Index score, developed by the U.S. EPA, measures neighborhood walkability based on residential density, land-use mix, street network design, and access to destinations, along with demographic and employment characteristics. Scores range from 0 to 100, with higher values indicating greater location efficiency

## Data Availability

This publication was produced from raw data purchased from or provided by the New York State Department of Health (NYSDOH). However, the conclusions derived, calculations, and views expressed herein are those of the author(s) and do not reflect the conclusions or views of NYSDOH. NYSDOH, its employees, officers, and agents make no representation, warranty or guarantee as to the accuracy, completeness, currency, or suitability of the information provided here. Dr. Lee had full access to all data used in the study and takes responsibility for the integrity of the data and the accuracy of the analysis. Due to patient confidentiality and in accordance with the data use agreement, the data used for analysis will not be shared.

## Abbreviations

CDC: Centers for Disease Control and Prevention
CI: Confidence interval
ED: Emergency department
EPA: Environmental Protection Agency
OP: Outpatient
HOLC: Home Owners’ Loan Corporation
LST: Land surface temperature
NDVI: Normalized Difference Vegetation Index
NYC: New York City
NYS: New York State
NYS-DOH: New York State Department of Health
PM: Particulate matter
PPM: Parts per million
RR: Rate ratio
SPARCS: Statewide Planning and Research Cooperative System
UHI: Urban heat island
US: United States
