# Supplementary material for: Redlining, Urban Heat, and Climate Hazards: Impacts on Children’s Cardiometabolic Health
Source: J Urban Health. Author manuscript; Available in PMC 2026 Aug 26. (PMC13507901; doi:10.1007/s11524-026-01132-9)
Supplement: Supplementary file 1 [file NIHMS2203518-supplement-Supplementary_file.pdf]

Supplementary Information

Redlining, urban heat, and climate hazards: Impacts on children's cardiometabolic health

<u>Contents</u>:

**eFigure 1**	Schematic illustration of the independent and synergistic exposure to climate risks and urban-heat.	**Pg. 2**
**eTable 1**	Pediatric healthcare visit characteristics by redlining status in New York State for the urban heat analyses, 2018-2022.	**Pg. 3**
**eTable 2**	Independent effects of residing in redlined or high-climate-risk areas, stratified by pandemic periods.	**Pg. 4**
**eTable 3**	Independent effects of residing in redlined or high-urban-heat areas, stratified by pandemic periods.	**Pg. 8**
**eTable 4**	Joint effects of residing in redlined and high-climate risk or high-urban-heat areas, stratified by pandemic periods.	**Pg. 9**

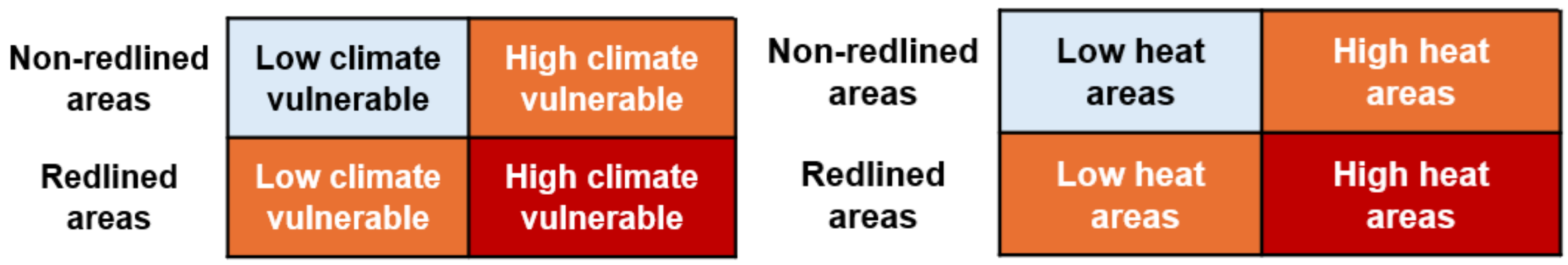

eFigure 1. Schematic illustration of the analytical framework for independent and synergistic exposures to climate risks and urban heat.

eTable 1. Pediatric healthcare visit characteristics by redlining status in New York State for the urban heat analyses, 2018-2022.

	Residential redlining status (by HOLC grades)[a]		
Characteristics	**All areas (A-D grades)** (n=9,165, 100%)	**Non-redlined areas (A/B grades)** (n=1,393; 15.2%)	**Redlined areas (C/D grades)** (n=7,772; 84.8%)
**Individual-level sociodemographic characteristics**			
Emergency department visits, No. (%)	1 105 (12.1)	212 (15.2)	893 (11.5)
Outpatient services, No. (%)	8 060 (87.9)	1 181 (84.8)	6 879 (88.5)
**Disease type**, No. (%)			
CV diseases, hyperlipidemia, comorbid[b,e]	794 (8.7)	140 (10.1)	654 (8.5)
Hypertension	695 (7.6)	174 (12.5)	521 (6.7)
Obesity	6 262 (68.3)	818 (58.7)	5 444 (70.0)
Type 1 & 2 Diabetes (T1D & T2D)[e]	1 414 (15.4)	261 (18.7)	1 153 (14.8)
**Race and ethnicity,** No. (%)			
Black, non-Hispanic	1 654 (18.0)	232 (16.7)	1 422 (18.3)
Hispanic, any race	3 598 (39.3)	461 (33.1)	3 137 (40.4)
White, non-Hispanic	960 (10.5)	301 (21.6)	659 (8.5)
Other, Asian, non-Hispanic, Unknown[c,d]	2 953 (32.2)	399 (28.6)	2 554 (32.9)
Age, mean (SD)	7.4 (5.9)	6.7 (5.9)	7.4 (5.9)
**Age group**, No. (%)			
Less than 5	3 775 (41.2)	608 (43.6)	3 167 (40.7)
5 to 9	1 760 (19.2)	260 (18.7)	1 500 (19.3)
10 to 14	2 088 (22.8)	299 (21.5)	1 789 (23.0)
15 to 17	1 542 (16.8)	226 (16.2)	1 316 (16.9)
**Sex,** No. (%)			
Female	4 493 (49.0)	678 (49.0)	3 815 (49.1)
Male	4 669 (50.9)	714 (51.0)	3 955 (50.9)
**Insurance coverage type,** No. (%)			
Private	3 789 (41.3)	554 (39.8)	3 235 (41.6)
Medicaid/CHP	1 942 (21.2)	267 (19.2)	1 675 (21.6)
Self-pay, Other/Unknown	3 434 (37.5)	572 (41.1)	2 862 (36.8)

Abbreviations: HOLC, Home Owners' Loan Corporation; CV, cardiovascular; CHP, Children's Health Plus

a. HOLC grades represent historical neighborhood classifications used in residential security maps: grade A (green) denotes "best" areas, grade B (blue) "still desirable," grade C (yellow) "declining," and grade D (red) "hazardous" areas.

b. Cardiovascular diseases included acute myocardial infarction, atrial fibrillation, congestive heart failure, stroke or transient ischemic attack, ischemic heart disease

c. Other race included American Indian or Alaska Native, Native Hawaiian or other Pacific Islander, or other race.

d. Unknown included any race classified without ethnicity.

e. As per the data use agreement, and to comply with the Centers for Medicare and Medicaid Services' small cell size policy, disease subtypes with values of 10 or fewer were combined. Similarly, Asian, other, and unknown race/ethnicity categories were combined for the same reason. Self-pay and other/unknown sources of payment were also combined.

eTable 2. Independent effects of residing in redlined or high-climate-risk areas, stratified by pandemic periods.

		Model 1[a] RR (95%CI)	Model 2[b] RR (95%CI)	Model 3[c] RR (95%CI)
**Study periods**				
**All periods (2018-2022)**	**Residence in redlined vs. non-redlined areas**			
	**Emergency department visits**			
	All CMDs	1.13 (1.10, 1.16)	1.15 (1.12, 1.18)	1.15 (1.12, 1.18)
	CVDs[d]	0.67 (0.55, 0.81)	0.74 (0.60, 0.91)	0.74 (0.60, 0.91)
	Comorbidity[e]	1.18 (0.96, 1.45)	1.35 (1.10, 1.67)	1.35 (1.10, 1.67)
	Hyperlipidemia	1.21 (1.06, 1.38)	1.20 (1.05, 1.36)	1.20 (1.05, 1.36)
	Hypertension	1.47 (1.37, 1.59)	1.56 (1.45, 1.68)	1.56 (1.45, 1.68)
	Obese	1.19 (1.14, 1.24)	1.22 (1.17, 1.27)	1.22 (1.17, 1.27)
	Type 1 diabetes (T1D)	0.96 (0.90, 1.03)	1.13 (1.05, 1.21)	1.13 (1.05, 1.21)
	Type 2 diabetes (T2D)	0.86 (0.80, 0.92)	0.89 (0.83, 0.96)	0.89 (0.83, 0.96)
	**Outpatient visits**			
	All CMDs	1.22 (1.20, 1.23)	1.18 (1.17, 1.20)	1.18 (1.17, 1.20)
	CVDs[d]	1.01 (0.93, 1.09)	1.02 (0.94, 1.10)	1.02 (0.94, 1.10)
	Comorbidity[e]	0.92 (0.86, 0.99)	0.93 (0.86, 1.00)	0.93 (0.86, 1.00)
	Hyperlipidemia	1.07 (1.02, 1.13)	0.95 (0.90, 1.00)	0.95 (0.90, 1.00)
	Hypertension	1.08 (1.03, 1.12)	1.26 (1.20, 1.31)	1.26 (1.20, 1.31)
	Obese	1.29 (1.28, 1.31)	1.24 (1.23, 1.26)	1.24 (1.23, 1.26)
	Type 1 diabetes (T1D)	1.17 (1.12, 1.21)	1.48 (1.42, 1.54)	1.48 (1.42, 1.54)
	Type 2 diabetes (T2D)	1.07 (1.01, 1.15)	1.02 (0.96, 1.09)	1.02 (0.96, 1.09)
	**Residence in high vs. low-climate risk areas**			
	**Emergency department visits**			
	All CMDs	0.95 (0.94, 0.96)	0.96 (0.95, 0.97)	0.95 (0.94, 0.96)
	CVDs[d]	0.88 (0.81, 0.94)	1.07 (0.98, 1.16)	1.12 (1.03, 1.23)
	Comorbidity[e]	1.05 (0.98, 1.13)	1.32 (1.23, 1.43)	1.31 (1.21, 1.41)
	Hyperlipidemia	0.87 (0.83, 0.91)	0.82 (0.78, 0.86)	0.81 (0.77, 0.85)
	Hypertension	0.96 (0.93, 0.99)	1.23 (1.19, 1.27)	1.20 (1.16, 1.23)
	Obese	0.99 (0.97, 1.00)	1.05 (1.03, 1.07)	1.04 (1.02, 1.06)
	Type 1 diabetes (T1D)	1.24 (1.23, 1.24)	1.09 (1.09, 1.10)	1.08 (1.08, 1.09)
	Type 2 diabetes (T2D)	0.97 (0.95, 1.00)	1.20 (1.17, 1.23)	1.20 (1.17, 1.23)
	**Outpatient visits**			
	All CMDs	1.02 (1.02, 1.02)	0.98 (0.97, 0.98)	0.96 (0.96, 0.97)
	CVDs[d]	0.79 (0.77, 0.82)	0.90 (0.87, 0.93)	0.89 (0.86, 0.92)
	Comorbidity[e]	1.00 (0.98, 1.03)	1.28 (1.24, 1.32)	1.30 (1.26, 1.34)
	Hyperlipidemia	0.97 (0.96, 0.99)	0.79 (0.78, 0.81)	0.79 (0.77, 0.80)
	Hypertension	0.78 (0.76, 0.79)	0.91 (0.90, 0.93)	0.88 (0.86, 0.90)
	Obese	1.08 (1.08, 1.09)	1.05 (1.05, 1.06)	1.04 (1.03, 1.04)
	Type 1 diabetes (T1D)	0.94 (0.93, 0.95)	1.15 (1.13, 1.17)	1.10 (1.08, 1.12)
	Type 2 diabetes (T2D)	0.80 (0.78, 0.81)	0.99 (0.96, 1.01)	0.99 (0.96, 1.01)
**Pre-pandemic (2018-2019)**	**Residence in redlined vs. non-redlined areas**			
	**Emergency department visits**			
	All CMDs	1.12 (1.07, 1.18)	1.15 (1.10, 1.21)	1.15 (1.10, 1.21)
	CVDs[d]	0.54 (0.38, 0.78)	0.61 (0.42, 0.89)	0.61 (0.42, 0.89)
	Comorbidity[e]	1.18 (0.95, 1.46)	1.17 (0.94, 1.46)	1.17 (0.94, 1.46)
	Hyperlipidemia	1.36 (1.08, 1.71)	1.17 (0.92, 1.49)	1.17 (0.92, 1.49)
	Hypertension	1.74 (1.54, 1.96)	1.77 (1.57, 1.99)	1.77 (1.57, 1.99)
	Obese	1.11 (1.09, 1.13)	1.06 (0.04, 1.08)	1.05 (1.03, 1.07)
	Type 1 diabetes (T1D)	0.89 (0.87, 0.91)	0.82 (0.79, 0.84)	0.80 (0.78, 0.83)
	Type 2 diabetes (T2D)	0.77 (0.68, 0.87)	0.77 (0.68, 0.87)	0.77 (0.68, 0.87)
	**Outpatient visits**			
	All CMDs	1.27 (1.24, 1.30)	1.26 (1.23, 1.29)	1.26 (1.23, 1.28)
	CVDs[d]	0.84 (0.74, 0.96)	0.88 (0.77, 1.01)	0.88 (0.77, 1.01)
	Comorbidity[e]	0.99 (0.92, 1.08)	0.99 (0.91, 1.07)	0.99 (0.91, 1.07)
	Hyperlipidemia	1.32 (1.17, 1.49)	1.36 (1.21, 1.54)	1.36 (1.21, 1.54)
	Hypertension	1.14 (1.05, 1.24)	1.35 (1.24, 1.46)	1.35 (1.24, 1.46)
	Obese	1.36 (1.32, 1.39)	1.35 (1.32, 1.39)	1.35 (1.32, 1.39)
	Type 1 diabetes (T1D)	1.21 (1.13, 1.30)	1.50 (1.40, 1.61)	1.50 (1.40, 1.61)

	Type 2 diabetes (T2D)	1.17 (1.01, 1.37)	1.04 (0.89, 1.22)	1.04 (0.89, 1.22)
	**Residence in high vs. low-climate risk areas**			
	**Emergency department visits**			
	All CMDs	0.95 (0.93, 0.97)	0.98 (0.96, 1.00)	0.97 (0.95, 0.99)
	CVDs[d]	0.96 (0.84, 1.10)	1.21 (1.02, 1.43)	1.34 (1.12, 1.60)
	Comorbidity[e]	1.09 (1.01, 1.18)	1.31 (1.21, 1.42)	1.31 (1.21, 1.42)
	Hyperlipidemia	0.86 (0.80, 0.93)	0.77 (0.71, 0.84)	0.77 (0.70, 0.84)
	Hypertension	1.07 (1.02, 1.12)	1.47 (1.39, 1.55)	1.44 (1.36, 1.52)
	Obese	0.99 (0.96, 1.02)	1.12 (1.08, 1.16)	1.10 (1.07, 1.14)
	Type 1 diabetes (T1D)	1.00 (0.96, 1.04)	1.26 (1.21, 1.32)	1.26 (1.20, 1.32)
	Type 2 diabetes (T2D)	0.80 (0.76, 0.84)	0.90 (0.86, 0.94)	0.92 (0.87, 0.97)
	**Outpatient visits**			
	All CMDs	1.01 (1.00, 1.02)	1.00 (0.99, 1.00)	0.98 (0.97, 0.98)
	CVDs[d]	0.75 (0.72, 0.79)	0.89 (0.84, 0.94)	0.89 (0.84, 0.95)
	Comorbidity[e]	1.03 (1.00, 1.06)	1.33 (1.29, 1.37)	1.34 (1.30, 1.39)
	Hyperlipidemia	0.86 (0.83, 0.90)	0.72 (0.69, 0.75)	0.69 (0.66, 0.72)
	Hypertension	0.76 (0.74, 0.79)	0.93 (0.90, 0.96)	0.89 (0.86, 0.92)
	Obese	1.06 (1.06, 1.07)	1.08 (1.07, 1.09)	1.06 (1.05, 1.07)
	Type 1 diabetes (T1D)	0.89 (0.87, 0.92)	1.00 (0.98, 1.03)	0.93 (0.90, 0.95)
	Type 2 diabetes (T2D)	0.70 (0.67, 0.74)	0.84 (0.79, 0.89)	0.84 (0.79, 0.89)
**During pandemic 2020-2022**	**Residence in redlined vs. non-redlined areas**			
	**Emergency department visits**			
	All CMDs	1.13 (1.09, 1.17)	1.14 (1.10, 1.18)	1.13 (1.10, 1.18)
	CVDs[d]	0.89 (0.69, 1.14)	0.98 (0.74, 1.29)	0.98 (0.74, 1.29)
	Comorbidity[e]	1.30 (0.62, 2.73)	5.05 (2.28, 11.2)	5.05 (2.28, 11.2)
	Hyperlipidemia	1.18 (1.00, 1.38)	1.26 (1.07, 1.48)	1.26 (1.07, 1.48)
	Hypertension	1.36 (1.24, 1.50)	1.46 (1.33, 1.61)	1.46 (1.33, 1.61)
	Obese	1.18 (1.12, 1.24)	1.20 (1.14, 1.26)	1.20 (1.14, 1.26)
	Type 1 diabetes (T1D)	0.96 (0.87, 1.05)	1.10 (1.00, 1.20)	1.10 (1.00, 1.20)
	Type 2 diabetes (T2D)	0.90 (0.83, 0.99)	0.97 (0.89, 1.06)	0.97 (0.89, 1.06)
	**Outpatient visits**			
	All CMDs	1.21 (1.19, 1.22)	1.16 (1.15, 1.18)	1.16 (1.15, 1.18)
	CVDs[d]	1.11 (1.01, 1.24)	1.10 (0.99, 1.22)	1.10 (0.99, 1.22)
	Comorbidity[e]	0.63 (0.50, 0.79)	0.67 (0.53, 0.84)	0.67 (0.53, 0.84)
	Hyperlipidemia	1.02 (0.96, 1.08)	0.84 (0.79, 0.89)	0.84 (0.79, 0.89)
	Hypertension	1.06 (1.01, 1.11)	1.23 (1.17, 1.29)	1.23 (1.17, 1.29)
	Obese	1.29 (1.27, 1.31)	1.22 (1.20, 1.24)	1.22 (1.20, 1.24)
	Type 1 diabetes (T1D)	1.14 (1.08, 1.19)	1.47 (1.40, 1.54)	1.47 (1.40, 1.54)
	Type 2 diabetes (T2D)	1.02 (0.95, 1.10)	1.04 (0.97, 1.12)	1.04 (0.97, 1.12)
	**Residence in high vs. low-climate risk areas**			
	**Emergency department visits**			
	All CMDs	0.95 (0.94, 0.96)	0.95 (0.94, 0.97)	0.94 (0.93, 0.95)
	CVDs[d]	0.87 (0.79, 0.95)	1.08 (0.97, 1.20)	1.10 (0.98, 1.23)
	Comorbidity[e]	0.92 (0.71, 1.18)	2.03 (1.45, 2.84)	1.63 (1.16, 2.30)
	Hyperlipidemia	0.87 (0.83, 0.92)	0.84 (0.79, 0.89)	0.83 (0.78, 0.88)
	Hypertension	0.90 (0.87, 0.93)	1.13 (1.08, 1.17)	1.09 (1.05, 1.14)
	Obese	1.01 (0.99, 1.03)	1.06 (1.04, 1.08)	1.05 (1.02, 1.07)
	Type 1 diabetes (T1D)	0.95 (0.92, 0.98)	1.16 (1.12, 1.20)	1.16 (1.12, 1.20)
	Type 2 diabetes (T2D)	0.84 (0.81, 0.86)	1.00 (0.96, 1.03)	1.00 (0.96, 1.03)
	**Outpatient visits**			
	All CMDs	1.04 (1.04, 1.05)	0.99 (0.98, 0.99)	0.97 (0.97, 0.98)
	CVDs[d]	0.82 (0.79, 0.84)	0.90 (0.87, 0.94)	0.89 (0.86, 0.93)
	Comorbidity[e]	0.88 (0.81, 0.96)	1.07 (0.97, 1.17)	1.11 (1.01, 1,22)
	Hyperlipidemia	1.00 (0.98, 1.02)	0.82 (0.80, 0.84)	0.82 (0.80, 0.84)
	Hypertension	0.79 (0.77, 0.80)	0.91 (0.90, 0.93)	0.89 (0.87, 0.91)
	Obese	1.11 (1.11, 1.12)	1.06 (1.06, 1.07)	1.05 (1.04, 1.06)
	Type 1 diabetes (T1D)	0.96 (0.95, 0.98)	1.23 (1.21, 1.25)	1.20 (1.17, 1.22)
	Type 2 diabetes (T2D)	0.80 (0.78, 0.82)	1.00 (0.98, 1.03)	1.00 (0.97, 1.03)

Abbreviations: RR, rate ratio; CI, confidence intervals; CMD, cardiometabolic disorders; CVD, cardiovascular diseases

[a] Model 1 is unadjusted model

[b] Model 2 is adjusted for age, sex, and race/ethnicity

[c] Model 3 is adjusted for Model 2 + census-tract level poverty rate, vehicle access, hospital proximity, local homicide and gun violence rates, food insecurity prevalence and healthy food access, proximity to high-traffic roads (vehicles per meter within 500 m), industrial and treatment/storage/disposal facilities (TSDFs), diesel particular matter, walkability, cooling centers, green space

[d] Cardiovascular (CV) diseases included: acute myocardial infarction, atrial fibrillation, congestive heart failure, stroke or transient ischemic attack, ischemic heart disease

[e] Co-morbidities include children who had two or more cardiometabolic-related co-morbidities

[f] Represent values with statistical significance alpha < .05; To facilitate visualization and interpretation, rate ratios were highlighted in yellow to represent statistically significant values, and in turquoise to represent increased risk values that did not reach statistical significance.

eTable 3. Independent effects of residing in redlined or high-urban-heat areas, stratified by pandemic periods.

Study periods		Model 1[a] RR (95%CI)	Model 2[b] RR (95%CI)	Model 3[c] RR (95%CI)
**All periods (2018-2022)**	<u>**Residence in redlined vs. non-redlined areas**</u>			
	**Emergency department visits**			
	All CMDs	1.03 (0.87, 1.21)	1.00 (0.85, 1.18)	1.00 (0.85, 1.18)
	CVDs[d]	1.01 (0.37, 2.82)	1.12 (0.38, 3.28)	1.12 (0.38, 3.28)
	Comorbidity[e]	0.48 (0.06, 3.68)	0.45 (0.06, 3.44)	0.45 (0.06, 3.44)
	Hyperlipidemia	0.93 (0.39, 2.20)	0.91 (0.38, 2.19)	0.91 (0.38, 2.19)
	Hypertension	1.05 (0.71, 1.56)	0.93 (0.62, 1.39)	0.93 (0.62, 1.39)
	Obese	1.01 (0.79, 1.31)	1.01 (0.78, 1.31)	1.01 (0.78, 1.31)
	Type 1 diabetes (T1D)	1.04 (0.72, 1.52)	1.16 (0.78, 1.73)	1.16 (0.78, 1.73)
	Type 2 diabetes (T2D)	0.97 (0.66, 1.43)	0.86 (0.58, 1.27)	0.86 (0.58, 1.27)
	**Outpatient visits**			
	All CMDs	1.02 (0.95, 1.09)	1.03 (0.96, 1.10)	1.03 (0.96, 1.10)
	CVDs[d]	0.90 (0.60, 1.33)	0.93 (0.62, 1.39)	0.93 (0.62, 1.39)
	Comorbidity[e]	0.85 (0.51, 1.42)	0.93 (0.56, 1.55)	0.93 (0.56, 1.55)
	Hyperlipidemia	1.13 (0.83, 1.53)	1.23 (0.90, 1.67)	1.23 (0.90, 1.67)
	Hypertension	0.91 (0.74, 1.12)	0.88 (0.72, 1.09)	0.88 (0.72, 1.09)
	Obese	1.06 (0.97, 1.16)	1.05 (0.96, 1.15)	1.05 (0.96, 1.15)
	Type 1 diabetes (T1D)	1.10 (0.90, 1.34)	1.28 (1.04, 1.57)[f]	1.28 (1.04, 1.57)[f]
	Type 2 diabetes (T2D)	1.03 (0.71, 1.49)	1.01 (0.69, 1.46)	1.01 (0.69, 1.46)
	<u>**Residence in high vs. low-urban heat areas**</u>			
	**Emergency department visits**			
	All CMDs	0.99 (0.87, 1.12)	1.00 (0.87, 1.13)	1.00 (0.87, 1.13)
	CVDs[d]	0.77 (0.31, 1.90)	0.91 (0.33, 2.47)	0.92 (0.33, 2.53)
	Comorbidity[e]	1.23 (0.44, 3.38)	1.19 (0.42, 3.32)	1.10 (0.38, 3.20)
	Hyperlipidemia	0.86 (0.45, 1.64)	0.83 (0.43, 1.60)	0.83 (0.43, 1.60)
	Hypertension	1.05 (0.71, 1.56)	0.93 (0.62, 1.39)	0.93 (0.62, 1.39)
	Obese	0.91 (0.74, 1.11)	0.91 (0.74, 1.11)	0.90 (0.74, 1.11)
	Type 1 diabetes (T1D)	0.94 (0.70, 1.25)	0.99 (0.74, 1.33)	1.00 (0.74, 1.34)
	Type 2 diabetes (T2D)	1.16 (0.85, 1.58)	1.14 (0.84, 1.55)	1.13 (0.83, 1.54)
	**Outpatient visits**			
	All CMDs	1.02 (0.97, 1.07)	1.02 (0.97, 1.08)	1.02 (0.97, 1.08)
	CVDs[d]	0.95 (0.67, 1.34)	0.96 (0.68, 1.36)	0.97 (0.67, 1.40)
	Comorbidity[e]	1.02 (0.73, 1.43)	1.05 (0.75, 1.47)	1.05 (0.75, 1.47)
	Hyperlipidemia	0.98 (0.78, 1.23)	1.04 (0.82, 1.30)	1.04 (0.82, 1.30)
	Hypertension	0.85 (0.71, 1.03)	0.81 (0.68, 0.98)[f]	0.82 (0.68, 0.99)[f]
	Obese	1.02 (0.96, 1.09)	1.02 (0.96, 1.08)	1.02 (0.96, 1.08)
	Type 1 diabetes (T1D)	1.21 (1.04, 1.42)[f]	1.27 (1.09, 1.49)[f]	1.24 (1.06, 1.46)[f]
	Type 2 diabetes (T2D)	1.09 (0.83, 1.45)	1.11 (0.83, 1.47)	1.11 (0.83, 1.48)
**Pre-pandemic (2018-2019)**	<u>**Residence in redlined vs. non-redlined areas**</u>			
	**Emergency department visits**			
	All CMDs	1.15 (0.87, 1.52)	1.16 (0.87, 1.55)	1.16 (0.87, 1.55)
	CVDs, comorbidity, hyperlip[d]	0.33 (0.10, 1.07)	0.43 (0.13, 1.41)	0.43 (0.13, 1.41)
	Hypertension	1.25 (0.67, 2.34)	1.15 (0.61, 2.16)	1.15 (0.61, 2.16)
	Obese	1.27 (0.79, 2.04)	1.25 (0.77, 2.03)	1.25 (0.77, 2.03)
	Diabetes	1.06 (0.68, 1.67)	1.11 (0.70, 1.75)	1.11 (0.70, 1.75)
	**Outpatient visits**			
	All CMDs	0.93 (0.81, 1.06)	0.94 (0.83, 1.08)	0.94 (0.83, 1.08)
	CVDs, comorbidity, hyperlip[d]	1.05 (0.71, 1.54)	1.11 (0.75, 1.64)	1.11 (0.75, 1.64)
	Hypertension	0.99 (0.70, 1.41)	1.04 (0.73, 1.47)	1.04 (0.73, 1.47)
	Obese	0.80 (0.67, 0.95)[f]	0.84 (0.70, 1.00)	0.84 (0.70, 1.00)
	Diabetes	1.06 (0.66, 1.68)	0.93 (0.58, 1.49)	0.93 (0.58, 1.49)
	<u>**Residence in high vs. low-urban heat areas**</u>			
	**Emergency department visits**			
	All CMDs	0.99 (0.80, 1.23)	1.02 (0.82, 1.26)	1.01 (0.81, 1.26)
	CVDs, comorbidity, hyperlip[d]	0.80 (0.43, 1.50)	0.85 (0.45, 1.59)	0.77 (0.40, 1.48)
	Hypertension	1.09 (0.63, 1.91)	1.12 (0.64, 1.96)	1.10 (0.63, 1.94)
	Obese	1.04 (0.71, 1.52)	1.02 (0.70, 1.50)	0.99 (0.67, 1.46)
	Diabetes	1.02 (0.73, 1.43)	1.08 (0.77, 1.51)	1.09 (0.77, 1.53)
	**Outpatient visits**			

	All CMDs	0.91 (0.83, 0.99)[f]	0.92 (0.83, 1.00)	0.92 (0.84, 1.01)
	CVDs, co-morbidity, hyperlip[d]	0.79 (0.61, 1.03)	0.79 (0.60, 1.03)	0.79 (0.61, 1.03)
	Hypertension	0.75 (0.55, 1.02)	0.73 (0.54, 0.99)[f]	0.72 (0.53, 0.99)[f]
	Obese	0.67 (0.59, 0.75)[f]	0.68 (0.61, 0.77)[f]	0.69 (0.61, 0.78)[f]
	Diabetes	0.82 (0.59, 1.14)	0.85 (0.61, 1.18)	0.84 (0.61, 1.17)
**During pandemic 2020-2022**	**Residence in redlined vs. non-redlined areas**			
	**Emergency department visits**			
	All CMDs	0.99 (0.82, 1.21)	0.94 (0.77, 1.15)	0.94 (0.77, 1.15)
	CVDs, comorbidity, hyperlip[d]	1.17 (0.55, 2.47)	1.03 (0.48, 2.22)	1.03 (0.48, 2.22)
	Hypertension	1.02 (0.62, 1.66)	0.89 (0.54, 1.47)	0.89 (0.54, 1.47)
	Obese	0.92 (0.68, 1.23)	0.92 (0.68, 1.25)	0.92 (0.68, 1.25)
	Diabetes	1.04 (0.74, 1.46)	0.97 (0.68, 1.37)	0.97 (0.68, 1.37)
	**Outpatient visits**			
	All CMDs	1.05 (0.97, 1.14)	1.06 (0.97, 1.15)	1.06 (0.97, 1.15)
	CVDs, comorbidity, hyperlip[d]	0.96 (0.73, 1.25)	1.00 (0.76, 1.31)	1.00 (0.76, 1.31)
	Hypertension	0.99 (0.76, 1.28)	0.96 (0.74, 1.25)	0.96 (0.74, 1.25)
	Obese	1.11 (0.99, 1.23)	1.09 (0.98, 1.21)	1.09 (0.98, 1.21)
	Diabetes	1.09 (0.90, 1.32)	1.19 (0.98, 1.45)	1.19 (0.98, 1.45)
	**Residence in high vs. low-urban heat areas**			
	**Emergency department visits**			
	All CMDs	1.00 (0.86, 1.17)	1.00 (0.85, 1.17)	1.00 (0.85, 1.17)
	CVDs, comorbidity, hyperlip[d]	1.13 (0.59, 2.19)	1.09 (0.55, 2.16)	1.10 (0.55, 2.20)
	Hypertension	1.20 (0.79, 1.81)	1.17 (0.77, 1.77)	1.18 (0.78, 1.79)
	Obese	0.88 (0.69, 1.12)	0.88 (0.69, 1.12)	0.88 (0.69, 1.12)
	Diabetes	1.07 (0.83, 1.39)	1.07 (0.83, 1.39)	1.07 (0.83, 1.39)
	**Outpatient visits**			
	All CMDs	1.06 (0.99, 1.12)	1.07 (1.00, 1.13)[f]	1.06 (1.00, 1.13)[f]
	CVDs, comorbidity, hyperlip[d]	0.98 (0.78, 1.21)	1.03 (0.83, 1.28)	1.03 (0.83, 1.28)
	Hypertension	0.91 (0.72, 1.14)	0.88 (0.70, 1.11)	0.88 (0.70, 1.11)
	Obese	1.05 (0.98, 1.13)	1.05 (0.98, 1.13)	1.05 (0.98, 1.12)
	Diabetes	1.27 (1.09, 1.48)[f]	1.33 (1.14, 1.56)[f]	1.31 (1.12, 1.53)[f]

Abbreviations: RR, rate ratio; CI, confidence intervals; CMD, cardiometabolic disorders; CVD, cardiovascular diseases

[a] Model 1 is unadjusted model

[b] Model 2 is adjusted for age, sex, and race/ethnicity

[c] Model 3 is adjusted for Model 2 + census-tract level poverty rate, vehicle access, hospital proximity, local homicide and gun violence rates, food insecurity prevalence and healthy food access, proximity to high-traffic roads (vehicles per meter within 500 m), industrial and treatment/storage/disposal facilities (TSDFs), diesel particular matter, walkability, cooling centers, green space

[d] Cardiovascular (CV) diseases included: acute myocardial infarction, atrial fibrillation, congestive heart failure, stroke or transient ischemic attack, ischemic heart disease

[e] Co-morbidities include children who had two or more cardiometabolic-related co-morbidities

[f] Represent values with statistical significance alpha < .05. To facilitate visualization and interpretation, rate ratios were highlighted in yellow to represent statistically significant values, and in turquoise to represent increased risk values that did not reach statistical significance.

eTable 4. Joint effects of residing in redlined and high-climate risk or urban-heat areas, stratified by pandemic periods.

		Model 1[a] RR (95%CI)	Model 2[b] RR (95%CI)	Model 3[c] RR (95%CI)
**Study periods**				
**All periods (2018-2022)**	**Interactions: redlining * high climate risk**			
	**Emergency department visits**			
	All CMDs	1.04 (1.01, 1.07)	1.04 (1.01, 1.07)	1.04 (1.01, 1.07)
	CVDs[d]	0.71 (0.59, 0.86)	0.76 (0.61, 0.94)	0.76 (0.61, 0.94)
	Comorbidity[e]	0.87 (0.68, 1.10)	0.89 (0.72, 1.11)	0.89 (0.72, 1.11)
	Hyperlipidemia	0.94 (0.83, 1.05)	0.88 (0.78, 0.98)	0.88 (0.78, 0.98)
	Hypertension	1.08 (1.01, 1.16)	1.04 (0.96, 1.11)	1.04 (0.96, 1.11)
	Obese	1.16 (1.12, 1.21)	1.14 (1.10, 1.19)	1.14 (1.10, 1.19)
	Type 1 diabetes (T1D)	0.86 (0.80, 0.92)	0.90 (0.84, 0.96)	0.90 (0.84, 0.96)
	Type 2 diabetes (T2D)	0.85 (0.79, 0.91)	0.92 (0.86, 0.98)	0.92 (0.86, 0.98)
	**Outpatient visits**			
	All CMDs	0.90 (0.89, 0.91)	0.92 (0.91, 0.93)	0.92 (0.91, 0.93)
	CVDs[d]	1.19 (1.10, 1.29)	1.32 (1.21, 1.43)	1.32 (1.21, 1.43)
	Comorbidity[e]	1.09 (1.00, 1.17)	1.15 (1.06, 1.24)	1.15 (1.06, 1.24)
	Hyperlipidemia	0.76 (0.73, 0.80)	0.77 (0.74, 0.81)	0.77 (0.74, 0.81)
	Hypertension	1.07 (1.03, 1.12)	0.99 (0.95, 1.03)	0.99 (0.95, 1.03)
	Obese	0.92 (0.90, 0.93)	0.94 (0.93, 0.95)	0.94 (0.93, 0.95)
	Type 1 diabetes (T1D)	0.79 (0.76, 0.83)	0.74 (0.71, 0.77)	0.74 (0.71, 0.77)
	Type 2 diabetes (T2D)	1.02 (0.96, 1.08)	1.16 (1.09, 1.23)	1.16 (1.09, 1.23)
	**Interactions: redlining * high urban heat**			
	**Emergency department visits**			
	All CMDs	1.15 (0.83, 1.59)	1.08 (0.78, 1.49)	1.08 (0.78, 1.49)
	CVDs[d]	0.91 (0.11, 7.24)	0.71 (0.09, 5.73)	0.71 (0.09, 5.73)
	Comorbidity[e]	NA[g]	NA[g]	NA[g]
	Hyperlipidemia	1.73 (0.30, 10.0)	2.31 (0.37, 14.3)	2.31 (0.37, 14.3)
	Hypertension	1.73 (0.78, 3.83)	1.61 (0.72, 3.58)	1.61 (0.72, 3.58)
	Obese	1.05 (0.63, 1.75)	1.00 (0.60, 1.67)	1.00 (0.60, 1.67)
	Type 1 diabetes (T1D)	1.25 (0.59, 2.68)	1.14 (0.53, 2.44)	1.14 (0.53, 2.44)
	Type 2 diabetes (T2D)	1.18 (0.52, 2.65)	1.02 (0.45, 2.32)	1.02 (0.45, 2.32)
	**Outpatient visits**			
	All CMDs	0.84 (0.73, 0.97)	0.83 (0.72, 0.96)	0.83 (0.72, 0.96)
	CVDs[d]	1.04 (0.41, 2.64)	0.89 (0.35, 2.26)	0.89 (0.35, 2.26)
	Comorbidity[e]	0.74 (0.26, 2.12)	0.87 (0.30, 2.52)	0.87 (0.30, 2.52)
	Hyperlipidemia	1.07 (0.58, 1.96)	1.10 (0.60, 2.00)	1.10 (0.60, 2.00)
	Hypertension	1.01 (0.66, 1.55)	0.89 (0.58, 1.37)	0.89 (0.58, 1.37)
	Obese	0.74 (0.62, 0.88)	0.72 (0.60, 0.86)	0.72 (0.60, 0.86)
	Type 1 diabetes (T1D)	1.11 (0.74, 1.67)	1.10 (0.73, 1.64)	1.10 (0.73, 1.64)
	Type 2 diabetes (T2D)	0.66 (0.32, 1.39)	0.63 (0.30, 1.33)	0.63 (0.30, 1.33)
**Pre-pandemic (2018-2019)**	**Interactions: redlining * high climate risk**			
	**Emergency department visits**			
	All CMDs	1.01 (0.97, 1.06)	1.02 (0.97, 1.07)	1.02 (0.97, 1.07)
	CVDs[d]	0.25 (0.17, 0.38)	0.61 (0.37, 1.00)	0.61 (0.37, 1.00)
	Comorbidity[e]	1.06 (0.83, 1.37)	1.10 (0.88, 1.38)	1.10 (0.88, 1.38)
	Hyperlipidemia	1.05 (0.85, 1.31)	1.06 (0.84, 1.34)	1.06 (0.84, 1.34)
	Hypertension	1.17 (1.04, 1.31)	1.20 (1.07, 1.35)	1.20 (1.07, 1.35)
	Obese	1.08 (1.00, 1.17)	1.06, 0.99, 1.15)	1.06 (0.99, 1.15)
	Type 1 diabetes (T1D)	0.83 (0.75, 0.93)	0.88 (0.79, 0.98)	0.88 (0.79, 0.98)
	Type 2 diabetes (T2D)	0.81 (0.73, 0.90)	0.86 (0.77, 0.96)	0.86 (0.77, 0.96)
	**Outpatient visits**			
	All CMDs	0.89 (0.87, 0.91)	0.90 (0.88, 0.91)	0.90 (0.88, 0.91)
	CVDs[d]	1.05 (0.92, 1.19)	1.31 (1.14, 1.50)	1.31 (1.14, 1.50)
	Comorbidity[e]	1.08 (1.00, 1.18)	1.15 (1.06, 1.25)	1.15 (1.06, 1.25)
	Hyperlipidemia	0.85 (0.76, 0.95)	0.84 (0.74, 0.94)	0.84 (0.74, 0.94)
	Hypertension	1.05 (0.97, 1.14)	0.94 (0.87, 1.02)	0.94 (0.87, 1.02)
	Obese	0.87 (0.85, 0.90)	0.87 (0.85, 0.90)	0.87 (0.85, 0.90)
	Type 1 diabetes (T1D)	0.55 (0.51, 0.60)	0.48 (0.45, 0.52)	0.48 (0.45, 0.52)
	Type 2 diabetes (T2D)	1.44 (1.25, 1.67)	1.60 (1.37, 1.86)	1.60 (1.37, 1.86)
	**Interactions: redlining * high urban heat**			
	**Emergency department visits**			

	All CMDs	1.24 (0.70, 2.18)	1.22 (0.69, 2.16)	1.22 (0.69, 2.16)
	CVDs, comorbidity, hyperlip[d,e]	NA[g]	NA[g]	NA[g]
	Hypertension	1.27 (0.35, 4.52)	1.04 (0.29, 3.76)	1.04 (0.29, 3.76)
	Obese	1.30 (0.48, 3.55)	1.23 (0.45, 3.38)	1.23 (0.45, 3.38)
	Diabetes	2.15 (0.87, 5.27)	1.99 (0.81, 4.90)	1.99 (0.81, 4.90)
	**Outpatient visits**			
	All CMDs	0.76 (0.59, 0.99)	0.73 (0.56, 0.96)	0.73 (0.56, 0.96)
	CVDs, comorbidity, hyperlip[d,e]	0.83 (0.39, 1.80)	0.87 (0.40, 1.89)	0.87 (0.40, 1.89)
	Hypertension	1.54 (0.71, 3.34)	1.17 (0.54, 2.53)	1.17 (0.54, 2.53)
	Obese	0.54 (0.38, 0.76)	0.48 (0.34, 0.69)	0.48 (0.34, 0.69)
	Diabetes	0.92 (0.35, 2.36)	0.99 (0.38, 2.56)	0.99 (0.38, 2.56)
**During pandemic 2020-2022**	**Interactions: redlining * high climate risk**			
	**Emergency department visits**			
	All CMDs	1.06 (1.02, 1.09)	1.06 (1.03, 1.10)	1.06 (1.03, 1.10)
	CVDs[d]	1.11 (0.85, 1.45)	1.06 (1.03, 1.10)	1.06 (1.03, 1.10)
	Comorbidity[e]	0.15 (0.06, 0.37)	0.38 (0.15, 0.95)	0.38 (0.15, 0.95)
	Hyperlipidemia	0.87 (0.75, 1.00)	0.80 (0.70, 0.92)	0.80 (0.70, 0.92)
	Hypertension	1.04 (0.95, 1.14)	0.97 (0.89, 1.07)	0.97 (0.89, 1.07)
	Obese	1.21 (1.15, 1.27)	1.19 (1.14, 1.26)	1.19 (1.14, 1.26)
	Type 1 diabetes (T1D)	0.90 (0.82, 0.99)	0.92 (0.83, 1.01)	0.92 (0.83, 1.01)
	Type 2 diabetes (T2D)	0.87 (0.80, 0.95)	0.93 (0.85, 1.02)	0.93 (0.85, 1.02)
	**Outpatient visits**			
	All CMDs	0.89 (0.88, 0.90)	0.92 (0.91, 0.93)	0.92 (0.91, 0.93)
	CVDs[d]	1.29 (1.16, 1.43)	1.26 (1.22, 1.51)	1.26 (1.22, 1.51)
	Comorbidity[e]	1.12 (0.88, 1.41)	1.21 (0.96, 1.52)	1.21 (0.96, 1.52)
	Hyperlipidemia	0.74 (0.70, 0.78)	0.77 (0.73, 0.82)	0.77 (0.73, 0.82)
	Hypertension	1.08 (1.03, 1,13)	1.00 (0.96, 1.05)	1.00 (0.96, 1.05)
	Obese	0.93 (0.92, 0.94)	0.96 (0.95, 0.97)	0.96 (0.95, 0.97)
	Type 1 diabetes (T1D)	0.94 (0.89, 0.99)	0.90 (0.86, 0.95)	0.90 (0.86, 0.95)
	Type 2 diabetes (T2D)	0.88 (0.83, 0.94)	1.02 (0.95, 1.09)	1.02 (0.95, 1.09)
	**Interactions: redlining * high urban heat**			
	**Emergency department visits**			
	All CMDs	1.09 (0.73, 1.61)	1.01 (0.68, 1.49)	1.01 (0.68, 1.49)
	CVDs, comorbidity, hyperlip[d,e]	1.81 (0.37, 8.73)	1.55 (0.30, 7.91)	1.55 (0.30, 7.91)
	Hypertension	1.73 (0.62, 4.82)	1.68 (0.59, 4.72)	1.68 (0.59, 4.72)
	Obese	0.96 (0.53, 1.74)	0.90 (0.50, 1.62)	0.90 (0.50, 1.62)
	Diabetes	1.02 (0.51, 2.07)	0.93 (0.45, 1.90)	0.93 (0.45, 1.90)
	**Outpatient visits**			
	All CMDs	0.97 (0.82, 1.15)	0.96 (0.82, 1.13)	0.96 (0.82, 1.13)
	CVDs, comorbidity, hyperlip[d,e]	1.21 (0.70, 2.09)	1.18 (0.68, 2.04)	1.18 (0.68, 2.04)
	Hypertension	1.00 (0.58, 1.72)	0.94 (0.54, 1.61)	0.94 (0.54, 1.61)
	Obese	0.88 (0.71, 1.09)	0.86 (0.70, 1.07)	0.86 (0.70, 1.07)
	Diabetes	1.01 (0.68, 1.49)	1.01 (0.68, 1.49)	1.01 (0.68, 1.49)

Abbreviations: RR, rate ratio; CI, confidence intervals; CMD, cardiometabolic disorders; CVD, cardiovascular diseases; NA, not applicable

[a] Model 1 is unadjusted model

[b] Model 2 is adjusted for age, sex, and race/ethnicity

[c] Model 3 is adjusted for Model 2 + census-tract level poverty rate, vehicle access, hospital proximity, local homicide and gun violence rates, food insecurity prevalence and healthy food access, proximity to high-traffic roads (vehicles per meter within 500 m), industrial and treatment/storage/disposal facilities (TSDFs), diesel particular matter, walkability, cooling centers, green space

[d] Cardiovascular (CV) diseases included: acute myocardial infarction, atrial fibrillation, congestive heart failure, stroke or transient ischemic attack, ischemic heart disease

[e] Co-morbidities include children who had two or more cardiometabolic-related co-morbidities; CVDs, comorbidities, and high cholesterol were sometimes analyzed together due to small sample sizes and the potential for overfitting.

[f] Represent values with statistical significance alpha < .05. To facilitate visualization and interpretation, rate ratios were highlighted in yellow to represent statistically significant values, and in turquoise to represent increased risk values that did not reach statistical significance.

[g] Because of the small number of counts, the result was null, possibly as a consequence of overfitting.
